# Clinical Trials in High-Risk Medulloblastoma: Evolution of the SIOP-Europe HR-MB Trial

**DOI:** 10.3390/cancers14020374

**Published:** 2022-01-13

**Authors:** Simon Bailey, Nicolas André, Lorenza Gandola, Maura Massimino, Keith Wheatley, Simon Gates, Victoria Homer, Stefan Rutkowski, Steven C. Clifford

**Affiliations:** 1 Great North Children’s Hospital, Queen Victoria Road, Newcastle upon Tyne NE1 4LP, UK; 2 Wolfson Childhood Cancer Research Centre, Newcastle University Centre for Cancer, Newcastle University, Newcastle upon Tyne NE1 7RU, UK; steve.clifford@newcastle.ac.uk; 3 Pediatric Hematology and Oncology Department, Hôpital Pour Enfants de La Timone, AP-HM, 13005 Marseille, France; Nicolas.ANDRE@ap-hm.fr; 4 Centre de Recherche en Cancérologie de Marseille, SMARTc Unit, Inserm U1068, Aix Marseille University, 13005 Marseille, France; 5 Pediatric Radiotherapy Unit, Fondazione IRCCS Istituto Nazionale dei Tumori, 20133 Milan, Italy; lorenza.gandola@gmail.com; 6 Pediatric Unit, Fondazione IRCCS Istituto Nazionale dei Tumori, 20133 Milan, Italy; maura.massimino@istitutotumori.mi.it; 7 Cancer Research UK Clinical Trials Unit, University of Birimingham, Birmingham B15 2TT, UK; k.wheatley@bham.ac.uk (K.W.); s.gates@bham.ac.uk (S.G.); v.s.homer@bham.ac.uk (V.H.); 8 Department of Pediatric Hematology and Oncology, University Medical Center Hamburg-Eppendorf, 20246 Hamburg, Germany; s.rutkowski@uke.de

**Keywords:** medulloblastoma, high-risk medulloblastoma, trial, CNS, brain tumour

## Abstract

**Simple Summary:**

Patients with medulloblastoma receive treatment according to a risk stratification, which is a combination of clinical and biological factors. To date there have been a limited number of trials for high-risk disease in children older than 3 years, with a wide range of treatment philosophies that usually involve higher doses of radiotherapy delivered either conventionally or in hyper-fractionated/accelerated regimens. Similarly, both standard and high-dose chemotherapies were assessed. However, to date, trials in high-risk medulloblastoma have commonly been institutional or national, based on modest cohort sizes, and have not evaluated the relative performance of different strategies in a randomised fashion. We describe the concepts and design of the SIOP-E high-risk medulloblastoma clinical trial (SIOP-HR-MB), the first international, biomarker-driven, randomised clinical trial for high-risk medulloblastoma. SIOP-HR-MB is programmed to recruit >800 patients in 16 countries across Europe; its primary objectives are to assess the relative efficacies of the alternative established regimens.

**Abstract:**

Medulloblastoma patients receive adapted therapies stratified according to their risk-profile. Favourable, standard, and high disease-risk groups are each defined by the status of clinical and pathological risk factors, alongside an evolving repertoire of diagnostic and prognostic biomarkers. Medulloblastoma clinical trials in Europe are coordinated by the International Society for Paediatric Oncology (SIOP-Europe) brain tumour group. Favourable and standard-risk patients are eligible for the SIOP-PNET5-MB clinical trial protocol. In contrast, therapies for high-risk disease worldwide have, to date, encompassed a range of different treatment philosophies, with no clear consensus on approach. Higher radiotherapy doses are typically deployed, delivered either conventionally or in hyper-fractionated/accelerated regimens. Similarly, both standard and high-dose chemotherapies were assessed. However, trials to date in high-risk medulloblastoma have commonly been institutional or national, based on modest cohort sizes, and have not evaluated the relative performance of different strategies in a randomised fashion. We describe the concepts and design of the SIOP-E high-risk medulloblastoma clinical trial (SIOP-HR-MB), the first international biomarker-driven, randomised, clinical trial for high-risk medulloblastoma. SIOP-HR-MB is programmed to recruit >800 patients in 16 countries across Europe; its primary objectives are to assess the relative efficacies of the alternative established regimens. The HR-MB patient population is molecularly and clinically defined, and upfront assessments incorporate a standardised central review of molecular pathology, radiology, and radiotherapy quality assurance. Secondary objectives include the assessment of (i) novel therapies within an upfront ‘window’ and (ii) therapy-associated neuropsychology, toxicity, and late effects, alongside (iii) the collection of materials for comprehensive integrated studies of biological determinants within the SIOP-HR-MB cohort.

## 1. High-Risk Medulloblastoma: Background, Challenges, and Basis for Clinical Trials

Medulloblastoma is the most common malignant brain tumour in children and young people, with approximately 650 new cases per year in the European Union (EU). These small, round, blue cell tumours of the posterior fossa account for 15–20% of all brain tumours in children. The median age of diagnosis is 7 years, but medulloblastoma occurs at all ages and into adulthood. The following variants of medulloblastoma are recognised in the World Health Organisation (WHO) classification of central nervous system (CNS) tumours (2016 and 2021) [1,2].

*Medulloblastoma, Genetically Defined*  

1. Medulloblastoma—WNT-activated;
2. Medulloblastom—SHH-activated and *TP53*-mutant;
3. Medulloblastoma—SHH-activated and *TP53*-wildtype;
4. Medulloblastoma—non-WNT/non-SHH (encompassing Group 3 and Group 4).

*Medulloblastoma, Histologically Defined*  

1. Classic medulloblastoma;
2. Desmoplastic/nodular medulloblastoma;
3. Medulloblastoma with extensive nodularity;
4. Large-cell/anaplastic medulloblastoma.

Our understanding of these variants and their clinical relevance is evolving and altering our understanding of prognosis and risk, creating a shifting scope of disease stratification [3,4,5]. Around 30% of MB patients are diagnosed as high-risk; currently defined clinically by the presence of one or more of the following high-risk factors: metastatic disease (i.e., M+), large cell/anaplastic (LCA) histology, *MYC* or *MYCN* amplification or significant residual disease post-surgery (i.e., R+).

High-risk medulloblastoma is associated with a 5-year, event-free survival (EFS) of about 60% [6,7,8,9,10,11]. Moreover, those patients that are cured have significant long-term toxicities (including neurocognitive and endocrinological toxicities) [12,13,14]. The median intelligence quotient (IQ) following therapy for medulloblastoma is in the order of 80 with significant effects on processing speed. This effect shows a linear relationship with a dose of radiotherapy (RT), memory and concentration, in addition to endocrinological, neurological, ototoxic and nephrotoxic effects, which have a significant effect on the quality of the rest of the patient. In many cases, independent living will not be possible and the ability to hold down a job unlikely. Initial studies indicate the severity of toxicity and late effects may be associated with the treatment received, clinico-biological disease features, and host genetic factors [7,12].

There is an urgent need to improve survival in patients with high-risk medulloblastoma, whilst at the same time, limiting acute and long-term toxicities that have a significant and detrimental impact on the quality of life of survivors. It is also vital to undertake the biological analysis of tumour samples to identify (i) those patients currently defined as having high-risk disease, but who have a better prognosis and may be better treated as standard-risk patients, and (ii) those patients who are unlikely to be cured by current conventional therapy, and/or in whom the evaluation of novel therapies at an earlier stage may be appropriate.

## 2. Definition of High-Risk Medulloblastoma: Trial Eligibility and Therapy Considerations

In current practice, high-risk disease is defined by the age of the patient, the presence of metastasis (Chang stages M1–M4; M+) and the amount of residual disease left following surgical resection (>1.5 cm^2^; R+). Histological, and now biological, factors refine the definition of risk. LCA pathology, tumour *TP53* mutation (in sonic hedgehog (SHH) subgroup tumours) and *MYC* or *MYCN* amplification are all used as high-risk factors to exclude patients from standard-risk protocols. Furthermore, it is now accepted that wingless-type (WNT) subgroup tumours in patients under the age of 16 have a favourable prognosis [15]. Other favourable and poor prognostic subgroups are emerging, but are not yet clinically established; further studies are now required to consolidate these.

### 2.1. Metastatic Disease

Approximately 30% of medulloblastoma patients present with metastatic disease [1] and have a poorer prognosis. There is a clear worse outcome for image-defined intracranial disease dissemination (Chang stage M2) or spread to the spine (Chang Stage M3), but microscopic spread to the cerebrospinal fluid (CSF) (Chang stage M1) is independent of the presence of macroscopic metastasis [16,17]. Chang stages M1-M4 are thus considered high-risk [18]. Multicentre trials showed a significant rate of false staging of patients; described as not having metastasis on local imaging reports but revised on central review; this is reflected in a lower-than-expected survival in patients not undergoing central review [19,20]. Quality assurance thus mandates central review for all patients in this trial. M4 disease is exceptionally rare and the approach to its management must be individualised.

### 2.2. Histological Variants

The recognised histological variants of medulloblastoma in the WHO classification of CNS tumours (2016 and 2021) are as described above: classic medulloblastoma, desmoplastic/nodular medulloblastoma, medulloblastoma with extensive nodularity and large cell/anaplastic medulloblastoma [2]. Current treatment strategies use histology as a tool for risk-stratification. LCA medulloblastoma, although briefly classified separately as large-cell and anaplastic medulloblastoma, have now been re-grouped as one entity in the WHO 2016 classification due to the difficulty in differentiating these rare variants, which often show mixed phenotypes [1]. Large-cell medulloblastoma is characterised by predominant monomorphic cells with large, round vesicular nuclei, single prominent nucleoli and variable amounts of eosinophilic cytoplasm [21]. Highly aggressive behaviour has been described in several reports [22,23]. Severe cytological anaplasia is also recognised to be a negative prognostic factor [24].

### 2.3. Surgical Resection

The extent of resection is still currently considered as a prognostic variable in medulloblastoma when overt metastatic disease is excluded by initial staging; however, its influence on PFS and OS is not clear. Apart from the CCG-921 trial, undertaken in the pre-magnetic resonance imaging (MRI) era, there are roughly an equal number of studies that identify, or fail to identify, an association between the increased extent of resection and OS. In the biggest randomised trial so far reported for non-metastatic medulloblastoma patients by Packer et al. in 2006, the 15 patients with post-operative residual disease did not have a significantly worse prognosis than the others [20]. In the St Jude medulloblastoma-96 trial the “high” risk group represented by those six patients with only residual disease (non-metastatic) reported having 100% EFS/OS [7]. The presence of residual post-operative disease was prognostic in the SIOP PNET 4 trial [25], but more recent prognostic analyses of 184 medulloblastoma cases treated with HIT (German-speaking countries cooperative group) protocols did not reveal a role of residual disease in a multivariate evaluation [26]. In an analysis of 125 consecutive patients in a single Italian institution, the eight children with only residual disease did not have a statistically different EFS and OS from the patients without residual disease [27]. A recent report from the Paediatric Oncology Group (POG) 9631 protocol, exploring the role of concomitant oral etoposide during craniospinal irradiation, once again did not find residual disease as prognostic factor [28]. Furthermore, it is probable that the prognostic benefit of a total resection is attenuated after accounting for a molecular subgroup affiliation [4].

Considering all of these data, there is a paucity of supportive evidence that intensifying therapy to the craniospinal axis improves local control in the setting of subtotal resection. Presently, the SIOP-E group recommends that a residual tumour without any other high-risk factors should be treated similarly to standard-risk disease.

### 2.4. Molecular Biomarkers

The discovery of molecular disease subgroups represents the most fundamental recent advance in our biological understanding of medulloblastoma. The current international consensus recognises four subgroups—WNT, SHH, Group 3 and Group 4 [29] and further subtypes within these subgroups were recently described [3,5,30,31]. Each subgroup is defined empirically by genome-wide transcriptomic and DNA methylation patterns [5,32] and characterised by distinct clinico-pathological and molecular features. WNT and SHH are synonymous with WNT (wnt/wingless pathway) and SHH (sonic hedgehog pathway)-activating mutations, respectively [33,34]. Childhood WNT patients (<16 years at diagnosis) consistently show a favourable prognosis (>90% survival) [7,35,36,37]. In addition, significant biological heterogeneity is evident within each non-WNT subgroup, for instance, *TP53* mutations associate with a poor outcome in SHH [5,38]. The loss of p53 function is thought to confer resistance to chemotherapy [39,40], and effective anti-tumoural treatments have yet to be established for this group, which represents approximately 10 patients in Europe per year. In contrast, Group 3 and Group 4 harbour few mutations but multiple DNA copy number alterations [34]. Importantly, subgrouping and *TP53* status are now integral to the World Health Organization (WHO) MB classification and are considered the ‘standard-of-care’ [2]. In addition to WNT- and SHH/*TP53*-mutated tumours, the presence of *MYC* or *MYCN* amplification were consistently identified as independent prognostic factors in trials-based studies [14,35,40]. *MYC/MYCN* amplification is also significantly associated with metastasis and LCA histology [41]. Schema that incorporate these combined factors significantly outperform risk-stratification using clinical factors alone [4,35]. The prognostic significance of *MYC/MYCN* amplification and histology is likely to be relevant only in the context of molecular subgrouping (e.g., *MYC* amplification in Group 3 tumours; *MYCN* amplification in SHH but not Group 4 tumours); therefore, clearer risk groups may become apparent as these refined prognostic associations are validated [5,42].

*MYCN* amplification was considered a high-risk factor in the original SIOP-PNET5-MB protocol, based on its association with a poor prognosis in studies undertaken across the disease prior to the identification of the four consensus molecular subgroups [35,41]. Two large retrospective studies have since been undertaken which assessed the prognostic impact of *MYCN* amplification with reference to these subgroups [5,42]. In both studies, *MYCN* amplification was associated with the SHH and Group 4 subgroups and displayed different clinical outcomes in each. In SHH, *MYCN* amplification was associated with a poor prognosis and commonly co-occurred with other high-risk factors (LCA pathology, *TP53* mutation, M+ disease). In contrast, *MYCN* amplification in Group 4 was not associated with a worse prognosis. These associations have since been validated in investigations of two groups of standard-risk patients (i.e., M− and R− with classic or desmoplastic pathology and no evidence of *MYC* amplification) from the HIT-SIOP-PNET4 clinical trial cohort and a UK research cohort [4,43].

Finally, emerging biological risk factors have the clear potential to further understand disease heterogeneity and improve the stratification of risk in medulloblastoma (e.g., novel molecular subgroups and/or whole-chromosome aberration patterns within Group 3/4 tumours [3,5,30,31,32] M+ in Group 4 tumours [4]). These require urgent evaluation and/or validation in the clinical trials setting, alongside biomarker discovery studies that focus on understanding heterogeneity within the high-risk medulloblastoma clinical group.

### 2.5. Familial/Germline Disease

Familial disease/germline mutations describe a notable proportion of medulloblastomas (5–10%); predominantly Gorlin (*PTCH1/SUFU* mutation in SHH patients), Turcot (Adenomatous-polyposis-coli *(APC)* in WNT patients), Li-Fraumeni (*TP53* in SHH patients) and Fanconi’s Anaemia (*BRCA2/PALB2*, subgroup unknown); they are associated with systemic radio- and chemosensitivity and must also be considered in therapy selection [30].

Although SHH subgroup patients with somatic *TP53* mutations are treated on high-risk protocols, chemotherapy-related toxicity and secondary malignancies are of great concern in patients with germline *TP53* mutations [44]. Alkylating drugs especially seem to exert a high geno-toxic stress in *TP53*-deficient backgrounds [45]. In a historic cohort of *n* = 37 patients with SHH-activated, germline *TP53*-mutated medulloblastoma treated with surgery, chemotherapy and radiotherapy, 3- and 5-year EFS were 20% and 16%, respectively, and no long-term survivors were detected (Milde, personal communication). No difference in OS and PFS was detected when patients were treated with chemotherapy before RT as compared to RT immediately after surgery, suggesting that chemotherapy before radiotherapy (i.e., a delay of radiotherapy) does not significantly influence the outcome. Thus, there is currently no consensus on the treatment of SHH-activated, germline *TP53*-mutated medulloblastoma patients, and specific clinical studies are required for this patient group.

Children and young people currently eligible for trials of high-risk medulloblastoma are summarised in Table 1.

## 3. Treatments for High-Risk Medulloblastoma and Future Potential

Prior to the 1990s, outcomes for high-risk medulloblastoma were poor, with 5-year EFS < 50% [17,46,47,48,49]. To improve survival, regimens looked to intensify treatment, either by increasing the dose of radiation, and through approaches including the use of high-dose or intensive chemotherapy, stem-cell rescue, or radiosensitisers. Since then, there have been several national or institutional trials that achieved 5-year EFS rates of around 60% (summarised in Table 2) [6,7,8,9,10,11]. The approaches used are dependent on national or institutional trials experience and include (i) high-dose chemotherapy prior to (or occasionally post-) craniospinal RT [6,7,8], (ii) HART (twice daily) [7,10,49], and (iii) conventional craniospinal RT (once daily), most commonly prior to maintenance chemotherapy [9,10].

Recent improvements in outcomes for patients with high-risk medulloblastoma are related to the systematic use of intensive chemotherapy regimens, including stem-cell rescue and the delivery of increasing doses of irradiation [6,7,8,9,10,11,50]. The improvement of modern radiotherapy techniques contributed to these clinical results, ensuring a more precise dose coverage of the whole neuraxis, reducing the risk of underdosage, and thus of the risk of relapse [51]. The gold standard radiotherapy for high-risk medulloblastoma, as described in the most recent clinical trials, is considered to be the delivery of craniospinal irradiation at a dose of 36–39.6 Gy with a conventional fractionation of 1.8–2 Gy per fraction, plus a boost up to 54 Gy to the primary site.

High-dose-intensity regimens, containing chemotherapy as well as radiotherapy, may result in an increase in significant long-term toxicities, particularly neurological and neurocognitive toxicities, as compared to less intensive regimens adopted for standard-risk medulloblastoma. However, in the most recent published series showing an increase in EFS, the impact of new, intensive treatment strategies, in particular high-dose cranio-spinal irradiation, on long term side-effects, including quality of life, was not assessed in detail.

Altered fractionation schedules of irradiation represent a possible approach to limit or reduce the impact of high-dose radiotherapy on the developing nervous tissue without compromising medulloblastoma control. The hyperfractionated-accelerated radiotherapy regimen (HART), as investigated by the Milan group [8], seems to be the most effective non-conventional schedule tested in the HRMB clinical setting. HART offers potential radiobiological advantages and was shown to be feasible in a UK study [50]. Hyperfractionation exploits the differences in repair capacity between normal and tumour cells and acceleration (larger doses per fraction, reduced length of treatment, hence increased treatment intensity); it has the potential to reduce tumour cell repopulation [52].

The Milan group showed that, in a prospective series of 33 children with metastatic medulloblastoma, HART, combined with sequential high-dose chemotherapy and consolidation myeloablative chemotherapy in selected cases, improved event-free survival (70% ± 8% standard error (SE) at 5 years) as compared with most historical series. In this single institution series, toxicity was acceptable considering the improved outcome, and it was detailed in two papers [53,55]. The HART regimen adopted, based on the linear quadratic model [56], was originally defined in the attempt to improve the therapeutic results without exacerbating the late sequelae of the conventional treatment, delivering 1.8 Gy daily fractions up to 36 Gy to the neuraxis and 54 Gy to the posterior fossa. Table 3 reports the extrapolated response dose for the tumour (ERD _T_) and for late-responding tissue (ERD _L_), according to the Dale equation, of the two schedules, HART and conventional fractionation (CF). As shown in Table 3, the HART regimen, increasing the dose intensity of irradiation, implies a potential improvement of radiotherapy efficacy in a tumour (ERD _T_) of about 5.8 and 4 points for CSI and tumour bed boost, respectively (ERD _T_ column) as compared to conventional fractionation, while the late response of normal tissue is substantially equivalent between the two radiotherapy modalities (ERD _L_ column).

## 4. Limitations in Recent Clinical Trials and Requirements for Future Studies

Patient cohorts examined to date have commonly been small (i.e., <50 patients) and often restricted to selected patient groups. In addition, the criteria for patient selection and risk stratification have varied over time and between studies. Moreover, the relative merits of the different approaches in current use have not been tested in a systematic way with respect to the heterogeneous disease biology we now appreciate, or in a large, randomised, multi-national trial to ascertain whether any of these strategies offer a survival advantage. No trials considered a biological stratification or subgroup analysis.

Importantly, high-risk medulloblastoma studies have not considered recent advances in our understanding of its biology. This is now fundamental to contemporary clinical research in medulloblastoma: First, contemporary molecular diagnostics are now essential to select and define the study population of clinical trials in high-risk medulloblastoma. As important are comprehensive, integrated, biological analyses of host and tumour features within the cohort and the assessment of their relationships to clinical outcomes, which will be essential to enable an improved understanding of the clinico-biological basis of high-risk medulloblastoma, its response to therapy, and impacts on the patient (e.g., toxicities and late effects). Finally, strategies for the development, stratification and assessment of novel therapies targeted against critical features of high-risk medulloblastoma will be essential to future advances. The consideration of these factors in future trials designs will, therefore, optimise opportunities to improve outcomes, and to develop future research and clinical trials concepts.

## 5. Evolution of Medulloblastoma Clinical Trials by the SIOP-Europe Group

### 5.1. SIOP-E and First Trials

The International Society for Paediatric Oncology (SIOP) was established in 1969 with the intention of promoting clinical trials of novel therapies in a wide range of children’s cancers. The European branch of SIOP (SIOP-E) and its Brain Tumour Committee demonstrated its capacity to deliver clinical trials by running the first two medulloblastoma trials, SIOP-1 and SIOP-2, in the 1970s and 1980s [56,57].

### 5.2. UKCCSG-SIOP-PNET3 (1993–2000)

The next SIOP-E medulloblastoma trial demonstrated a significant survival benefit of the addition of chemotherapy to adjuvant radiotherapy, provided tumour samples and patient cohorts for biological studies, and developed integral post-treatment quality-of-life studies as added measures [58,59]. In contrast to children without macroscopic metastases (M0/M1), pre-irradiation chemotherapy did not show apparent improvements in outcome for patients with macroscopic metastases (M2/3) when compared with earlier multi-institutional series [48].

### 5.3. HIT-SIOP-PNET4 (2000–2006)

This subsequent study assessed the relative benefits of standard and hyper-fractionated radiotherapy regimes in children with non-metastatic medulloblastoma from 9 European countries, demonstrating equivalent outcomes using these approaches [37]. LCA pathology was the only biological parameter used for stratification at that time, as this risk factor became a non-inclusion criterion through an amendment. HIT-SIOP-PNET4 continued the embedded SIOP-E principles of collecting tissues and survivorship data to support critical research co-studies [11,36,37].

### 5.4. First Biologically Driven Trials—SIOP-PNET5-MB (2014–2022)

UKCCSG-SIOP-PNET3 and HIT-SIOP-PNET4 permitted the investigation of tumour biology and its clinical impact on homogeneously treated trial cohorts. This establishment of bio-characterisation strategies within SIOP-E trials, complementing careful pathological, imaging and surgical staging systems, provided the critical framework for advances in prognostication, risk-stratification and risk-adapted treatment selection. UKCCSG-SIOP-PNET3 biological studies first identified the WNT subgroup and its favourable prognosis [32,57] and subsequently developed integrated schemes for the stratification of patients into three risk-groups using combined clinical, pathological and molecular factors: favourable-risk (WNT subgroup), high-risk (non-WNT tumours with M+, R+, LCA pathology or *MYC/MYCN* amplification) and standard-risk (all remaining patients) [3]. HIT-SIOP-PNET4 subsequently validated and refined risk stratification; limiting the favourable prognosis of WNT patients to those under 16 years at diagnosis and the poor prognosis of *MYCN* amplification to SHH subgroup patients, alongside the discovery of novel prognostic subgroups (i.e., favourable-risk, non-WNT/non-SHH patients characterised by a whole-chromosome aberration phenotype) for further investigation [36].

These schemes form the basis of patient selection and therapy selection for the current SIOP-E trial for children with favourable-risk and standard-risk medulloblastoma (SIOP-PNET5-MB; NCT 02066220). Favourable-risk patients (SIOP-PNET5-MB-LR) receive reduced-intensity chemo- and radiotherapy that aims to maintain survival rates while limiting therapy-associated late effects; standard-risk patients received the randomised addition of concomitant carboplatin (SIOP-PNET5-MB-SR). High-risk patients, identified through the criteria and the national real-time molecular diagnostics and pathology review systems established for SIOP-PNET5-MB [58], represent eligible candidates for trials of high-risk medulloblastoma; facilitating patient work-up for all trials using common pathways.

SIOP-E medulloblastoma trials, from SIOP-1 through to SIOP-PNET5-MB and SIOP-HR-MB, were all conducted for children older than 3–5 years at diagnosis. For their younger counterparts, specific SIOP-E trials are currently being developed for the first time (YCMB-LR and YCMB-HR).

## 6. The SIOP-Europe High-Risk Medulloblastoma Trial (SIOP-HR-MB)

SIOP-HR-MB opened to recruitment in February 2021, and is the largest trial of high-risk medulloblastoma patients undertaken to date, programmed to include 850 patients from 16 European countries. There is no upper age limit. A lower age limit of 3–5 years at diagnosis will be applied depending on national preferences. SIOP-HR-MB is a partner to the SIOP-PNET5-MB trial for standard- and favourable-risk medulloblastoma, utilising common diagnostic and central review pathways for molecular, pathological and radiological assessments.

SIOP-HR-MB will evaluate outcomes using differing chemotherapy and radiotherapy strategies for patients diagnosed with high-risk medulloblastoma. Eligible patients are defined as positive for any validated high-risk factor: M+, LCA pathology, *MYC* amplification, *MYCN* amplification or *TP53* somatic mutation (both in SHH subgroup tumours only). The collection of frozen and FFPE tumour tissue, and a blood sample, is mandated to support these pathways, which are now considered the standard of care for medulloblastoma [20,27,28].

## 7. Objectives

### 7.1. Primary Objectives

1. To evaluate whether the outcome in children, young people and adults with high-risk medulloblastoma is improved over standard therapy for those treated with; (i) conventional (once a day) radiotherapy (control arm), (ii) hyperfractionated/accelerated radiotherapy (HART), or (iii) high-dose therapy with thiotepa followed by conventional radiotherapy;
2. To evaluate whether the outcome for high-risk patients is different for those treated with two different maintenance chemotherapy therapies.

### 7.2. Secondary Objectives

1. To study the late effects of treatment and their impact on quality of survival (QoS), including neurocognitive function, neurological impairment, endocrine impairment, audiological function and secondary tumours;
2. To conduct comprehensive and prospective biological studies in high-risk medulloblastoma, with the aims of (i) understanding the biological basis of high-risk disease, (ii) identification and validation of diagnostic and prognostic biomarkers, and (iii) identification and validation of molecular targets with therapeutic potential and associated predictive biomarkers;
3. To conduct prospective QoS, toxicity and pharmacogenomic studies with the aim of exploring clinical, host and tumour factors, and genetic variants that relate to the early and late side effects of treatment and survival parameters.

**Figure 1:**
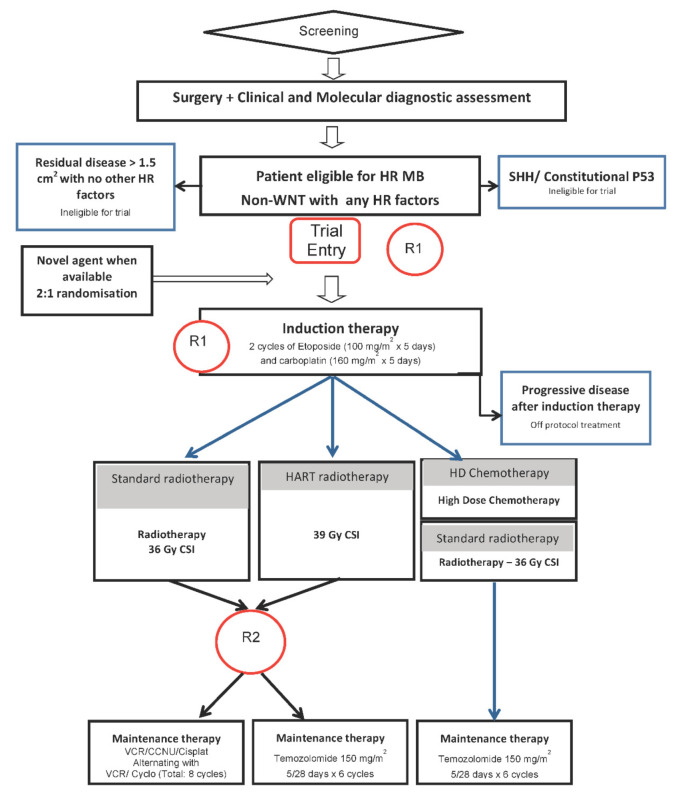
Schema for the SIOP-Europe high-risk medulloblastoma (SIOP-HR-MB) clinical trial. R1: Randomisation 1. To be performed at trial entry after screening. R2: Randomisation 2. To be performed after completion of radiotherapy and within 7 days prior to the planned start of maintenance therapy. Patients who are not eligible for further protocol therapy are advised to be treated in accordance with the current national guidelines.

The schema for SIOP-HR-MB is summarised in Figure 1.

If there is residual disease after initial surgery, further resection should be considered, but if not possible or feasible in the absence of other biological risk factors, suggested guidelines should be used which are outlined in this manuscript (line 636). Those with SHH group tumours and constitutional *TP53* mutation (routinely tested for in the initial biological workup) may enrol on PNET 5, where there is an arm specifically for these patients). For those that progress during initial chemotherapy craniospinal radiotherapy should be initiated and further chemotherapy is suggested in the relapsed/progressive disease section of the manuscript.

## 8. Trial Description

The current European standard treatment for high-risk medulloblastoma post-surgery is induction chemotherapy (usually carboplatin and etoposide) followed by conventional (once a day) radiotherapy (RT) at a dose of 36 Gy to the craniospinal axis with an additional boost to the posterior fossa, after which maintenance chemotherapy is given using a cisplatin, CCNU (lomustine), cyclophosphamide and vincristine-based therapy. The trial will assess alternative treatment approaches that may improve survival without significantly increasing toxicity. Given that high-risk medulloblastoma patients have a poor prognosis, intensification of treatment is justified to evaluate whether this reduces the risk of recurrence with an acceptable toxicity. Two ways in which this intensification can be achieved are by increasing either the dose of RT or the chemotherapy by giving high-dose therapy (HDT) with a stem cell rescue. Two experimental arms will therefore be compared with standard therapy in a randomised fashion (R1): (i) the current European standard treatment (control, as above), (ii) an arm based on the use of HART and (iii) an arm based on HDT [1,3,4,5,59]. For those randomised to receive high-dose chemotherapy, two cycles of high-dose thiotepa will be given at 600 mg/m^2^ (200 mg/m^2^ for 3 consecutive days), a minimum of 21 days following induction chemotherapy, followed by peripheral stem cell re-infusion. Two cycles will be administered with a 21-day interval between the commencement of course 1 and 2 [54].

HART at 39 Gy will be given to the neuraxis, two fractions per day, plus tumour bed boost (increased dose-intensity of radiotherapy) [8] and (iii) conventionally fractionated irradiation at 36 Gy to the neuraxis, plus tumour bed boost delivered after high-dose chemotherapy and stem cell rescue (increased dose-intensity of chemotherapy) [6]. It is well established that craniospinal irradiation represents the standard radiation treatment for medulloblastoma; this requires complex planning systems and delivery techniques to ensure the accurate coverage of the entire central nervous system. This aims to avoid disease relapse due to insufficient dosing in the case of high-risk disease requiring high craniospinal irradiation (CSI) doses, and at the same time, avoid the risk of severe damage to the neural tissue. There is a sparsity of published data concerning the role of focal radiotherapy to the primary tumour site, as well as to the metastatic sites of disease. With this in mind, this trial uses the following approaches:

1. Boost the primary tumour site to the tumour bed only, and not to the whole posterior fossa, considering the need to limit toxicity in view of the absence of data on the role of local irradiation after CSI in the setting of HR-MB patients. Central radiological review and the high-quality MRI required in the trial allow for a precise definition of tumour extension and the substantial sparing of normal nervous tissue related to the tumour bed boost, thereby allowing the safer delivery of boost doses to the brain metastatic sites when indicated.
2. Boost metastatic sites (if no more than three measurable lesions remain after induction chemotherapy), taking into account that the response to chemotherapy is considered a good prognostic factor [8,11], thus avoiding the need to boost mainly normal brain and spinal tissue without evidence of macroscopic disease, which is at high risk of significant side effects, such as radio-necrosis. Moreover, boosts to numerous metastatic sites would imply the delivery of very high doses to large volumes of the central nervous system without existing evidence on the efficacy of this approach but with a high probability of increasing toxicity.

A second, subsequent randomisation (R2) will compare standard intravenous (IV) maintenance chemotherapy with oral temozolomide. IV maintenance chemotherapy, however, is significantly toxic, with up to 56% of children requiring dose or drug modification. It is plausible that single-agent maintenance might be more effective or as effective with less toxicity. Temozolomide showed activity against MB, mainly in relapsed patients, both as a monotherapy, as well as in combination [54,60,61]. Promising results were reported by Dufour et al., using six cycles of oral temozolomide following high-dose chemotherapy in metastatic MB (5-year EFS 72%) [5]. Those patients who received HDT will not tolerate cisplatin, CCNU, cyclophosphamide and vincristine and will all receive temozolomide.

## 9. Central Review and Research Investigations

The central review of radiology and pathology, as well as biological investigations, share a common pathway with the SIOP-E standard and favourable-risk medulloblastoma trial, SIOP-PNET5-MB. Patients ineligible for SIOP-PNET-5 on molecular pathological or radiological review may represent eligible candidates for SIOP-HR-MB.

Practices developed through SIOP-PNET5-MB, which introduced standardised, real-time, centralised, molecular diagnostics and a pathology review for medulloblastoma patients across Europe, were applied to SIOP-HR-MB. These are supported by the routine collection of high-quality samples, i.e., fresh-frozen and FFPE tumour material, blood (all mandatory) and CSF (optional), essential for clinical and research investigations [43]. A biology and pathology group within the SIOP-E embryonal tumour group works to establish, undertake, coordinate and ensure the quality control of these processes [58], together with translational biological studies, within all SIOP-E medulloblastoma clinical trials; the committee has representatives from all partner countries.

### 9.1. Radiology and Radiotherapy

Central radiological review is undertaken prior to trial entry in each participating country, both pre- and post-operatively, with particular focus on residual and metastatic disease, prior to confirming trial eligibility. In addition, the central prospective review of the complex planning for CSI and tumour bed boost is undertaken to ensure consistent adherence to protocol. A detailed atlas for outlining the targets as well as normal organs at risk was published and is available as an online atlas to aid protocol adherence [62].

### 9.2. Biological Investigations: Reference Assessments and Biological Studies

The overall strategy for biological investigations within SIOP-HR-MB is two-fold; (i) to use molecular diagnostics of well-defined biomarkers to enrol and stratify patients into SIOP-HR-MB, and (ii) to conduct comprehensive studies on the biological basis of medulloblastoma, with the aim of the identification, investigation and validation of biomarkers and drug targets with potential to improve management of the disease.

### 9.3. Molecular Diagnostics

Centralised molecular diagnostics and a pathology review must be completed within 3 weeks post-surgery to enable timely planning and commencement of adjuvant therapies. The definition of diagnostic criteria for molecular tests, and quality control/validation of diagnostic methods, are essential components of the SIOP-E biology group’s work, and evolved to introduce emerging technologies and methods through protocol amendments. Critical advances included a requirement for the definition of molecular subgroup status by a consensus across at least two independent assays (e.g., immunohistochemistry (IHC), direct beta-catenin mutation analysis, DNA methylation or expression profiling), the definition of thresholds for the positivity of ‘gold-standard’ iFISH-based testing for *MYC* and *MYCN* amplification status, and the introduction of pathologist panels to review interpretation of histological variant and IHC analysis [36,58].

### 9.4. Biological Research

Following upfront diagnostic assessments, samples are shipped to designated international research coordinating centres (Newcastle University, Newcastle upon Tyne, UK (for all of Europe); Bonn (for Germany, Austria and Switzerland)). Here, frozen and FFPE tissues are processed, and tissue microarrays (TMAs) are constructed to support biological studies. Comprehensive biological studies are performed on surplus collected material by a network of partner research centres, to advance biological understanding of the disease, and identify and validate next prognostic and predictive biomarkers. A comprehensive core set of prospective biological investigations are undertaken (RNA-seq, Illumina-850K-copy number/DNA methylation, panel sequencing (tumour/germline) of all commonly mutated medulloblastoma genes), alongside the establishment of a tissue, TMA and DNA/RNA resource for future planned studies, such as WGS, proteomic and ctDNA (CSF) evaluations.

### 9.5. Neurocognition and Quality of Survival

Quality of survival (QOS) will be assessed on four occasions (post-surgery before induction therapy, at two and five years after diagnosis, and again at age 18 years) using several brief questionnaires, which include HUI, SDQ, PedsQL Core + Fatigue module, BRIEF and MEES. Neurocognitive assessments will be performed at the same time points using the core domains of neurocognitive functioning following the recommendations for participants aged 5 years and older enrolled in European childhood brain tumour trials based on Limond et al. [63], with an order of priority as agreed by the European QoS group in January 2018. Participants who are 4 years old at the time of assessment should be assessed using a similar, but not identical, battery of tests. For participants who are younger than 4 (3 years) at the time of assessment an indirect measure of development will be ascertained via the Vineland Adaptive Behaviour Scales (2nd edition or 3rd edition) questionnaire.

### 9.6. Key Research Questions

Integrated biological and genetic datasets obtained will be used, alongside clinical phenotyping, to address key questions and inform the planning of future studies, including:

- Identification and/or validation of independent prognostic biomarkers which are associated with disease course in high-risk medulloblastoma;
- Development of models for the optimal prediction of disease risk, using combined clinical, pathological and molecular indices, within the high-risk strata;
- Prioritisation of potential therapeutic targets, and associated predictive biomarkers, for further investigation and validation;
- Investigation of novel germline predisposition within the cohort;
- Investigation of associations with clinical factors such as imaging features, quality of survival, intellectual outcomes and toxicity measures.

## 10. Balancing Recruitment to the Randomisation Arms

Patient numbers will be balanced across the three R1 randomisation arms, by molecular subgroup and key prognostic factors (R+, M+, LCA, *MYCN* amplification (SHH only), *MYC* amplification, and sporadic *TP53* mutation (SHH only)), to ensure the equivalent representation of each feature (and combinations of features) in each arm.

## 11. Relapse Management and Introduction of Novel Therapies

Around 25% of patients with high-risk medulloblastoma progress during treatment or relapse after treatment. Recently, it was reported that both the nature and outcome of medulloblastoma at relapse depended not only on their biology, but also on the treatments received [64,65]. The previous receipt of cranio-spinal radiotherapy had a strong negative impact on outcome at relapse [50,62,63]. Patients who relapse after irradiation are incurable with salvage therapy, with very rare exceptions (less than 5% of those who relapse) [66]. The main goals for patients relapsing or progressing on treatment are: (i) Symptom control and a satisfactory quality of residual life (i.e., giving priority to oral routes of administration, longer home stays and normal living habits, rather than seeking higher radiological response rates at the expense of more severe toxicities and longer hospital stays). These may be achieved by established second-line strategies, and (ii) the accrual into phase 1–2 trials of novel therapies, where available.

The availability of new agents and new strategies are essential to improve outcomes of patients with relapsing/refractory medulloblastoma. Importantly, these treatments could be incorporated upfront in combination with current chemotherapies or during radiotherapy to potentially enhance the chance of a cure. New agents could also be incorporated in the maintenance phase of treatment during which the treatment is potentially less intensive, hence making it easier/safer to add new agents.

### 11.1. Potential New Agents and Approaches

New therapies can either rely on targeted therapies including drug repurposing, immunotherapies, or on well-known anti-cancer therapies given in a maximum tolerated dose (MTD) manner or in a metronomic fashion.

### 11.2. Targeted Therapies at Relapse

Recent findings indicated an increased involvement of pathways including DNA damage-signalling pathways, PI3K/mToR or/and CDK amplifications at relapse [67], and recent preclinical findings confirmed their therapeutic potential [68]. Nevertheless, the use of new anti-cancer agents was very limited at relapse, and the emergence of potential agents for medulloblastoma remains low. Thus, beyond SHH inhibition, no other targeted therapy is currently being used upfront or at relapse in children with medulloblastoma [33,69]. Elsewhere, targeting the *MYC* family of oncogenes could provide crucial steps forward for the most aggressive tumours. Strategies including BET [70] and Aurora kinase [65] inhibition showed pre-clinical potential in medulloblastoma [71]. High-throughput drug screening suggested that a combination of pemetrexed and gemcitabine could be a promising treatment for Group 3 medulloblastoma [72]; these form the basis of the SJ-ELIOT trial for medulloblastoma, which is currently underway (NCT04023669).

### 11.3. Immune Therapies

In children, immunotherapy relying on immune checkpoint inhibitors with antiPD1/PDL1 alone or in combination has not led to the clinical success observed in adults [73,74,75]. The development and progression of medulloblastoma are facilitated by a variety of immune-evading mechanisms including the secretion of immunosuppressive molecules, activation of immunosuppressive cells, inhibition of immune checkpoint molecules, impairment of adhesive molecules, downregulation of the MHC molecules, protection against apoptosis, and of course, the activation of immunosuppressive pathways. Understanding the tumour–immune relationship in medulloblastoma and its molecular subtypes will be crucial for the effective development of immune-based therapeutic strategies [76]. Chimeric antigen receptor therapies (CAR-T) are also under development for medulloblastoma and show promise [77].

### 11.4. Reuse of Standard Treatments

Reusing standard treatments, including re-irradiation, may also be a potential strategy. In a retrospective series of 24 patients, Baroniv [78] reported that the re-irradiation with CSI was both safe and effective for children with relapsed medulloblastoma. It contributed to the improved disease control and survival. Unfortunately, re-irradiation came at a high neurocognitive cost. Similar findings were reported by Gupta and Padovani et al. [79,80], who reported the combination of local re-irradiation and temozolomide to take advantage of both the radiosensitizing properties and the systemic effect of temozolomide in a small series of 5 patients [81]. The protection of the brain from radiation-induced injury or sensitization to radiation therapy might be achieved through drug repurposing with drugs such as lithium [82] or celecoxib [83].

### 11.5. Second-Line Chemotherapy

Grill and al. reported the potential of temozolomide and irinotecan in relapsed/refractory medulloblastoma [61]. Sixty-six patients were treated. The objective response rate during the first 4 cycles was 32%, with a median duration of response of 27 weeks (range: 7.7–44.1 weeks). Sixty-eight percent of the patients experienced a clinical benefit. The median survival was 16.7 months (95% CI, 13.3–19.8). The most common grade 3 treatment-related adverse event was neutropenia (16.7%). In a randomized phase 2 trial evaluating TEMIRI ± bevacizumab, the COG reported an increased median OS (13 months vs. 19 months), with the addition of bevacizumab among the 105 treated patients [84].

### 11.6. Metronomic Therapy

Metronomic chemotherapy in combination with drug repurposing is another potential strategy for patients with relapsing/refractory medulloblastoma [85]. Peyrl et al. [86] used an anti-angiogenic metronomic multidrug combination with bevacizumab, thalidomide, celecoxib, fenofibrate, etoposide, and cyclophosphamide and additional intraventricular therapy (etoposide and liposomal cytarabine). At the time of the report, 5 patients with MB were alive for 12, 33, 33, 37, and 58 months. One patient with medulloblastoma died in an accident after 23 months of treatment. A state-of-the-art international phase 2 trial is ongoing (NCT01356290). Other metronomic protocols reported interesting results [87]. Of note, metronomic chemotherapy could be introduced as a maintenance for high-risk medulloblastoma, as for other adults or paediatric malignancies [88].

## 12. Early Phase Window Concept

In addition to opportunities to assess therapies at relapse, SIOP-HR-MB planning incorporates a novel upfront window, prior to induction therapy, where therapeutics may be assessed. In this concept, agents with target-associated activity in medulloblastoma pre-clinical studies, and which showed promise in early phase trials, may be assessed. Target patient populations will be identified as part of upfront trial molecular diagnostics, and it is envisaged that multiple initial trials of different strategies could be developed over the lifetime of the SIOP-HR-MB.

## 13. Patients Not Eligible for the Trial

Those patients with SHH tumours and a *TP53* germline mutation will be excluded from the trial due to their very poor prognosis with conventional therapy. However, their patients are eligible for a purpose-designed arm in SIOP-PNET5-MB. Likewise, patients with R+ disease (after re-resection is considered), and with no other high-risk features, are not eligible for SIOP-HR-MB. The SIOP-E currently recommends that these patients receive carboplatin/etoposide induction therapy followed by a 23.4 Gy CSI plus boost to the posterior fossa (total 53.4 Gy), followed by maintenance therapy with cispslatin/CCNU/vincristine alternating with cyclophosphamide/VCR, as described above.

## 14. Conclusions

SIOP-HR-MB is the first pan-European trial for the treatment of high-risk medulloblastoma and tests, in a randomised fashion, three different treatment approaches; hyper-fractionated and accelerated radiotherapy, standard radiotherapy, and high-dose chemotherapy followed by standard radiotherapy. In addition, two alternative maintenance therapies are tested; CCNU/vincristine/cisplatin alternating with vincristine/cyclophosphamide and temozolomide only. The trial population is selected based on the upfront central assessment of clinical, pathological and molecular factors; radiological review and randomisation arms are balanced for key clinical and molecular risk factors. The flexible design includes an upfront window to allow for the assessment of novel/targeted therapies in selected groups and anticipates the future incorporation of emerging biological risk factors. Importantly, there is a common diagnostic pathway for favourable, standard and high-risk medulloblastoma across all SIOP-Europe medulloblastoma trials to ensure that patients are directed to the appropriate trial. The trial also forms the basis of focussed research to refine risk stratification, understand disease pathogenesis and late effects, and support the development of targeted therapies. The trial outcomes will inform the design of future trials.

## Figures and Tables

**Table 1:** Children older than 3 years at diagnosis are typically eligible for high-risk medulloblastoma trials based on current evidence. * Presence of at least one of these factors.

Molecular Features	Histology	Residual Tumour	Metastatic Disease
*TP53* mutant (somatic) and/or *MYCN* amplified (SHH subgroup only)	any	any	any
any non-WNT subgroup	LCA *	any	M+ *
WNT subgroup (>16 years)	LCA *	any	M+ *
*MYC* amplified (any subgroup)	any	any	any

**Table 2:** Summary of clinical trials in high-risk medulloblastoma. R+ = Residual disease > 1.5 cm^2^; M+ = metastatic disease; M1–3 = Chang metastatic staging.

Study [ref]	Number of Patients	Cohort Definition	Radiotherapy Dose	Chemotherapy	Comments	Toxic Deaths	Progression on Treatment	Event-Free Survival
SJMB96 [7]	48 (M0 = 6; M1 = 9; M2 = 6; M3 = 27)	R+ or M1–M3	36–39.6 Gy	4× HD chemotherapy (cisplatin, cyclophosphamide and vincristine) post-radiation	Single institute study; no randomization; part of a larger trial; 31/48 had additional pre-radiation topotecan window study. Quality of survival data published.	0	1	5-year EFS 70%
HART (UK) [50]	34 (M1 = 9; M2 = 3; M3 = 24)	M+	1.24 Gy fractions bd to 39.68 Gy	Vincristine with radiation Maintenance 8× cisplatin, CCNU, vincristine	Toxicity feasibility study/not powered for survival. Excluded patients requiring GA.	1	0	3-year EFS 59%
COG 99,701 [9]	161 (M0 = 5; M1 = 18; M2 = 10; M3 = 49)	R+, M+ or supratentorial PNET	36 Gy	Carboplatin and vincristine during radiation Maintenance with 6× cyclophosphamide and vincristine +/− cisplatin	Phase I/II carboplatin as radiosensitizer; no quality of survival data published.	0	4 (all long-term survivors, likely pseudo-progression)	5-year EFS M1 = 77% M2 = 50% M3 = 67%
POG 9031 [10]	224 (M1 = 29; M2 = 36; M3 = 34; M4 = 9)	T3b/T4, M+ or R+	35.2–40.0 Gy	Randomised 3x cisplatin and etoposide before or after radiation; Maintenance with 7× cyclophosphamide and vincristine	72 were Chang Stage T3b/T4, M0, R-; no quality of survival data published.	None reported	12 in the CT 1st arm	5-year EFS 66% CT 1st 70% RT 1st
Milan [8,53]	33 (M1 = 9; M2 = 6; M3 = 17; M4 = 1)	M+	HART 31.2–39 Gy	10 weeks chemotherapy pre-radiation (methotrexate, vincristine, etoposide, cyclophosphamide, carboplatin); post-radiation 2× HD chemotherapy (Thiotepa]) or maintenance with 12 months CCNU and vincristine	Limited centre study; Subsequent neuro toxicity reported. Quality of life data reported.	None reported	5 (pre-radiation) 2 (on maintenance therapy)	5-year EFS 70%
Institut Gustave Roussy (France) [6]	24 (M0 = 5; M1 = 0; M2 = 4; M3 = 15)	R+, M+, *MYCN* amplification or supratentorial PNET	18 Gy (1) 25 Gy (2) 36 Gy (19) 40 Gy (1) 54 Gy focal [1 sPNET]	2× carboplatin and etoposide pre-radiation; 2× HD chemotherapy (Thiotepa); Maintenance with temozolomide	Single institute study; neurocognitive data reported.	0	0	5-year EFS 65% 72% in M+
HIT 2000 (Germany) [11]	123 (M1 = 36; M2/M3 = 87)	M+	HFRT 40 Gy	2× cycles of pre-radiation chemotherapy (cyclophosphamide, vincristine, methotrexate, carboplatin, etoposide and intraventricular methotrexate); maintenance with 4 cycles cisplatin, CCNU, vincristine	Well-tolerated.	0	14 (pre-radiation) 1 (after radiation) 31 (during maintenance or at end of treatment)	5-year EFS 62%
PNET HR+5 (France) [54]	51 (M0 = 14; M1 = 3; M2/3 = 34)	R+, M+, *MYC/N* amplification, LCA histology	36 Gy CSI Unless Residual disease alone post surgery with no other high risk features then 23.4 Gy CSI	2× carboplatin/etoposide; 2× thiotepa HD; 6× temozolomide maintenance	French national study.			5-year EFS 76% 5-year OS 76%

**Table 3:** Extrapolated response dose for tumour (ERD T) and for late responding tissue (ERD L), according to the Dale equation, comparing HART and conventional fractionation (CF). HART = Hyperfractionated Accelerated Radiation Therapy; CF = Conventionally Fractionated radiotherapy.

RT Volume	Schedule	Total Dose	Dose/Fraction	Fractions/Day	No. Fractions	ERD _T_	ERD _L_
CSI	HART	39 Gy	1.3 Gy	2	30	31.47	55.9
	CF	36 Gy	1.8 Gy	1	20	25.68	57.6
Tumour Bed/Brain Metastasis boost	HART	20.8 Gy	1.3 Gy	2	16	16.78	29.8
	CF	18 Gy	1.8 Gy	1	10	12.84	28.8
Spine metastasis boost	HART	7.8 Gy	1.3 Gy	2	6	6.29	11.2
	CF	9 Gy	1.8 Gy	1	5	6.42	14.4
