# Correction: Bailey et al. Clinical Trials in High-Risk Medulloblastoma: Evolution of the SIOP-Europe HR-MB Trial. *Cancers* 2022, *14*, 374

**DOI:** 10.3390/cancers16061084

**Published:** 2024-03-07

**Authors:** Simon Bailey, Nicolas André, Lorenza Gandola, Maura Massimino, Keith Wheatley, Simon Gates, Victoria Homer, Stefan Rutkowski, Steven C. Clifford

**Affiliations:** 1 Great North Children’s Hospital, Queen Victoria Road, Newcastle upon Tyne NE1 4LP, UK; 2 Wolfson Childhood Cancer Research Centre, Newcastle University Centre for Cancer, Newcastle University, Newcastle upon Tyne NE1 7RU, UK; steve.clifford@newcastle.ac.uk; 3 Pediatric Hematology and Oncology Department, Hôpital Pour Enfants de La Timone, AP-HM, 13005 Marseille, France; nicolas.andre@ap-hm.fr; 4 Centre de Recherche en Cancérologie de Marseille, SMARTc Unit, Inserm U1068, Aix Marseille University, 13005 Marseille, France; 5 Pediatric Radiotherapy Unit, Fondazione IRCCS Istituto Nazionale dei Tumori, 20133 Milan, Italy; lorenza.gandola@gmail.com; 6 Pediatric Unit, Fondazione IRCCS Istituto Nazionale dei Tumori, 20133 Milan, Italy; maura.massimino@istitutotumori.mi.it; 7 Cancer Research UK Clinical Trials Unit, University of Birimingham, Birmingham B15 2TT, UK; k.wheatley@bham.ac.uk (K.W.); s.gates@bham.ac.uk (S.G.); v.s.homer@bham.ac.uk (V.H.); 8 Department of Pediatric Hematology and Oncology, University Medical Center Hamburg-Eppendorf, 20246 Hamburg, Germany; s.rutkowski@uke.de

## Missing Funding

In the original publication [1], the funder Cancer Research UK, A2524 was not included. 

## Addition of Authors

Keith Wheatley, Simon Gates, and Victoria Homer were not included as authors in the original publication. The reason we would like to add the authors is that the statistical element of the trial and the trial design were in a large part done by the statistical authors and the team were necessary for the running of the trial. The corrected Author Contributions Statement appears here.

**Author Contributions:** Conceptualization, S.B., N.A., L.G., M.M., K.W., S.R. and S.C.C.; methodology, K.W., S.G. and V.H.; project administration, S.G. and V.H.; resources, S.B., N.A., L.G., M.M., S.R. and S.C.C.; writing—original draft preparation, S.B., N.A., L.G., M.M., S.R. and S.C.C.; writing—review and editing, S.B., N.A., L.G., M.M., S.R. and S.C.C. All authors have read and agreed to the published version of the manuscript.

## Additional Affiliation

Cancer Research UK Clinical Trials Unit, University of Birimingham, Birmingham B15 2TT, UK; k.wheatley@bham.ac.uk(K.W.); s.gates@bham.ac.uk (S.G.); v.s.homer@bham.ac.uk (V.H.)

The authors apologize for any inconvenience caused and state that the scientific conclusions are unaffected. This correction was approved by the Academic Editor. The original publication has also been updated.
